# The Home Doctor

**Published:** 1883-09

**Authors:** 


### THE HOME DOCTOR.

Much may be done to prevent the croup by the exercise of good judgment in the care of children. Some parents make their children very sensitive and delicate by excessive prudence. A child kept in the house and treated like a house-plant can hardly be prevented from encountering a rude blast of wintry weather by the over-sight of some one having the care of it. It is better to toughen the child by clothing it warmly and give it a good airing every day, either in walking or riding. The mistake should not be made of putting fur or woolen about the throat. Toughen the throat as you do the face. Be sure that the extremities are as warmly clad as the trunk of the body. See to it that the shoes are made to exclude snow-water, and still avoid the use of rubbers, unless, if necessary, a pair of low sandals be used to protect the soles of the feet. One more hint to over-prudent mothers: Do not cover the child too warmly when put to bed. Nothing is more liable to give a child a cold than to be muffled in too many blankets or spreads of some kind, for as soon as it gets into an uncomfortable perspiration it will free itself from the cumbrous covering, and then by a sudden checking of the perspiration contract a cold. There is absolutely more danger from excessive than too little covering.—*Dr. Foote's Health Monthly.*

